# Teaching mode of oral English in the age of artificial intelligence

**DOI:** 10.3389/fpsyg.2022.953482

**Published:** 2022-07-22

**Authors:** Yun Li

**Affiliations:** School of Foreign Languages, Xinyang Normal University, Xinyang, China

**Keywords:** artificial intelligence, spoken English, teaching mode, natural language processing, oral ability

## Abstract

With the deepening of cultural integration, people’s demand for English learning is also increasing rapidly. However, traditional teaching methods have certain limitations, and teaching conditions are limited by the slow development of information technology, oral English courses have been shelved and stopped for a long time. With the rapid development of technology, the era of artificial intelligence has arrived. Learning assistance systems based on artificial intelligence have emerged in an endless stream, which has also innovatively solved the problem of oral language learning. Natural language processing is a computing mode of deep learning by artificial intelligence, which can carry out deep learning and training according to the current goal and finally get the desired result. But relying only on the auxiliary learning system cannot fundamentally solve the problem of oral language learning. Therefore, we aim to update the current spoken English learning methods using natural language processing technology, and propose a natural language processing-based oral English teaching model. In this mode, natural language processing can match different teaching methods according to the spoken language characteristics of different students, and give constructive suggestions. Moreover, the spoken English teaching mode based on natural language processing can be continuously upgraded and adjusted to adapt to the changing and developing era in time. Experiments show that the oral English teaching mode based on natural language processing can improve students’ comprehensive ability of oral English. And it increased its comprehension by 19.7% year-on-year, and at the same time it also improved the enthusiasm for learning oral language by 33.3%.

## Introduction

With the development of society, people are in urgent need of comprehensive English talents. Most importantly, spoken English is the most important of them all, and it is a direct indication of people’s English proficiency, which is why it has become a measure in English exams. However, in the actual teaching process in the classroom, due to the limitation of teaching conditions, teachers are often unable to carry out corresponding oral English courses. Natural language processing is one of the branches of artificial intelligence, which can simulate human processing of speech signals, so its introduction into the teaching mode of spoken English can greatly promote the renewal of teaching methods and models. Moreover, based on this technology, students’ current learning environment and English teaching mode will be completely new, which will greatly mobilize students’ enthusiasm and initiative in learning spoken English. Deep learning has brought major technological breakthroughs to natural language processing, and its wide application has greatly changed people’s daily lives. At the same time, the teaching mode based on natural language processing can not only give learners accurate and timely pronunciation evaluation and feedback guidance, but also help learners to find out the difference between their pronunciation and standard pronunciation, and correct pronunciation errors. It thus improves the learning efficiency of students’ spoken English, and takes this as an opportunity. It provides the student’s composite grade.

Teaching mode matching based on natural language processing can effectively improve students’ enthusiasm for learning oral language, allowing them to think at different levels and achieve progress. Among them, we can see that students’ enthusiasm for learning oral language has increased by 33.3% year-on-year, and their imitation ability has increased by 66.7%, which further promotes students to think actively and ask questions. After a period of pattern training and matching, the students’ speaking scores have been basically improved. Among them, in the final exam, the average score of the experimental class was 112.42, which was higher than the 112.13 of the control class. This shows that pattern matching based on natural language processing is beginning to work. But at the same time, we can also see that the standard deviation of the experimental class is much higher than that of the control class, which shows that the fluctuation of their scores is relatively large, and the dispersion of scores is relatively strong. At the end of the semester, we found that the experimental class’s performance improved significantly year-on-year, and the average scores of the two classes were higher than those of the control class. This fully shows that after a period of training and learning, the teaching mode based on natural language processing has achieved remarkable results.

## Related work

With the popularization of English learning, more and more teachers begin to study the teaching mode suitable for themselves and their students, in order to improve students’ enthusiasm for learning. At the same time, many experts and scholars have also turned their attention to this place.

Yin (2021) aimed to study the English embedded grammar supplemental pedagogy and apply it to the design of microclassroom model. He proposed a definition of micro-classroom and English grammar and related research on micro-classroom and English grammar teaching, as well as constructivist theory and hierarchical analysis, both of which will be used in the experimental part (Yin, 2021). Bahr et al. (2020) described three common patterns of spelling errors across specific learning disabilities, modes of presentation, and modes of transcription. And he discussed the pedagogical application of teaching students with specific learning disabilities to spell words in English while writing in the academic register (Bahr et al., 2020). Zeng (2021) studied a basic approach of flipped classroom, big data technology and neural network technology in a college English speaking class with the aim of verifying the effectiveness of the flipped classroom model, i.e., whether it can help college students improve their English speaking skills and their ability to learn on their own. The results of his experiment showed that the flipped classroom model and information technology led to a change in the traditional order of knowledge transfer and internalization by students. The flipped classroom focuses on students’ initiative to learn and assimilate knowledge before they start the course and then ask the teacher for help. His research validated the overwhelming advantages of the flipped classroom and proves that it is feasible in real life and teaching, making it an extremely promising and relatively new model of teaching spoken English (Zeng, 2021). Jiajia (2017) proposed a new teaching model of college English, which is mainly based on the computer network platform, and he analyzed the basic status and some problems of the traditional college English teaching model, and he also elaborated the overwhelming advantages and future development direction and prospect of his proposed teaching model. Based on this, he launched a large number of experiments and obtained many key data. The results show that information network technology has developed very rapidly and is widely used in various fields, thus leading to the reform of the teaching mode of college English in the direction of intelligence and informatization (Jiajia, 2017).

The above experts and scholars have proposed many English teaching models from different levels, but there are no methods and methods for oral English learning in the teaching models they put forward. Artificial intelligence can change the existing oral language teaching mode and teaching environment, so we study a series of literatures related to it.

Hassabis et al. (2017) believed that a better understanding of biological brains could play a crucial role in building intelligent machines. He therefore investigated the historical interaction between the fields of artificial intelligence and neuroscience, and highlights current advances in artificial intelligence. These advances are inspired by research on neural computing in humans and other animals (Hassabis et al., 2017). Makridakis’s (2017) main view was that the AI revolution will greatly change people’s production and life, and the coverage of this change is extremely wide. Furthermore, he found that the impact of this innovation on businesses and employment will be enormous, leading to increased global competition among highly interconnected organizations and businesses based on decision-making based on “big” data analysis and utilization. With the Internet, people can buy goods from all over the world (Makridakis, 2017). Rongpeng tried to emphasize the basic characteristics of technology in the 5 g era. In other words, all aspects of cellular network have the shadow of initial intelligence, such as the operation and management of radio and the supply and management of services (Li et al., 2017). Liu R attempted to give a comprehensive review of artificial intelligence algorithms in rotating machinery fault diagnosis from both theoretical background and industrial application. He started with his brief introduction to different artificial intelligence algorithms. He then surveyed the extensive literature on industrial applications of these AI algorithms. Finally, he discussed the advantages, limitations, practical implications, and some new research trends of different AI algorithms (Mao, 2022).

The above experts and scholars have extended artificial intelligence to different levels of society, focusing on analyzing the role of artificial intelligence in social life. However, it did not introduce the technology into the curriculum and teaching field, so its expansion is not very comprehensive.

## Artificial intelligence and oral English teaching mode

### English and spoken English

With the rapid arrival of globalization, English can be used all over the world. Moreover, it has also become one of the common languages, and it is very important for students to master the oral ability in the process of English learning. Because this will have a direct impact on its future development (Glauner et al., 2017). Therefore, the teaching of spoken English is very crucial for teachers, and they need to use a series of positive teaching methods. It can effectively improve students’ speaking ability and make students willing to practice their own speaking. It enables it to become a human resource useful to society.

But nowadays, there are some problems in the teaching of spoken English. These include the lack of suitable oral English teaching environment; the neglect of the learner’s dominant position, the old-fashioned teaching mode of oral English, etc. (Thrall et al., 2018). Specifically, the traditional concept of exam-oriented education is too deep-rooted, and teachers mainly focus on teaching materials. However, it is necessary for students to practice in a certain oral English environment to make progress. However, students feel very boring because of the cramming learning environment. Under the general environment, students’ oral English study is only to cope with the exam, and there is no need for real oral communication. In this case, students are caught in the dilemma of mechanical learning and memorization of spoken English, and they cannot really feel the great charm and importance of spoken English (Zhou and Wu, 2022). Therefore, it also has a negative impact on the teaching level of spoken English, and it is also a great obstacle to the students’ ability to use oral English.

In addition, the one-size-fits-all teaching model used by teachers also ignores the individual differences of each student. They blindly catch up with the teaching progress, while ignoring the students’ receptive ability, and the teacher’s pace of speaking class is too fast. Often, before the students have absorbed and digested the content of the previous class, the teacher will start to teach the next chapter, which puts the students in a very disadvantageous passive position. Some students have relatively weak learning ability, and it is difficult to catch up with the teacher’s progress. Therefore, these students will be more likely to lose confidence and motivation in oral English learning, they are afraid of taking oral English classes, and their ability will be difficult to improve and develop.

The way teachers teach also has a critical impact on student learning. If the teacher just repeats a certain knowledge without innovating the teaching method, the students will have a feeling of disgust for the oral students, and there is no enthusiasm for learning. On the contrary, they will have a rebellious mentality and are unwilling to participate in oral activities or competitions with a proactive attitude (Chen et al., 2017). Generally speaking, the teaching modes of spoken English are as follows (Nasr et al., 2017). First, students’ oral English foundation should be solidified, and at the same time, extracurricular teaching activities should be used to improve them. The learning of spoken language is not single, it needs to be supplemented by the training of reading ability and listening ability, so that the overall ability of English can be improved. In this learning process, students can accumulate a certain vocabulary, making their pronunciation more standard (Lemley et al., 2017). During the reading process, teachers can help students select reading materials that they are more interested in reading aloud, and divide students into different groups. It allows them to speak English loudly and emotionally in this environment, and it allows each student to fully demonstrate their talents. In addition, some English clubs or seminars in the school can also be a suitable environment for them to practice their spoken English.

**FIGURE 1:**
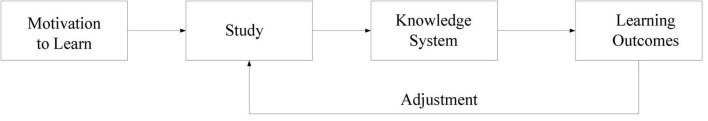
Learning structure diagram in fixed mode.

Second, students are the main body of learning, and their learning ability varies. Therefore, teachers should have a basic understanding of each student’s oral ability. And it follows the teaching principle of teaching students in accordance with their aptitude, and strives not to leave any students behind. According to the size of the students’ ability, they will be trained at different levels. If the ability is weak, start with relatively simple exercises, and then gradually increase the difficulty. In addition, the teaching method should not be too single, and it is necessary to adopt a more diversified teaching method, so as to attract students’ interest and attention (Price and Flach, 2017). Most importantly, teaching methods need to be adjusted according to changes in teaching content. By choosing more interesting activities for pre-class introduction, to stimulate their enthusiasm for learning and turn passive into active. Among them, the method of situational teaching is the most common. As the name suggests, this method refers to allowing students to integrate into a situation, and let them play corresponding roles and use spoken language to perform. One of the biggest advantages of this method is that it can quickly activate the atmosphere of the classroom, and at the same time, it can improve students’ ability to use spoken language. Among them, the learning structure diagram in the fixed mode is shown in Figure 1.

### Artificial intelligence

The full name of artificial intelligence in English is artificial intelligence, abbreviated as AI (Burton et al., 2017). At the same time, artificial intelligence is also a new technical science that studies and develops theories, methods, technologies and application systems for simulating, extending and expanding human intelligence. It refers to making the machines produced by people have the cognitive behavior, thinking ability and learning activities of the outside world like people, and simulate human thinking. Artificial intelligence is changing people’s production and life in its own unique way.

In the beginning, artificial intelligence was just a branch of computer science technology, but then it tried to understand the essence of intelligence and try to do things that humans take a lot of time to do (Agrawal et al., 2017). Artificial intelligence is extremely dependent on the development level and degree of computer technology, so its application field is initially limited to the recognition and input of text, speech, and images. It makes the research of AI have a lot of instability and uncertainty.

**FIGURE 2:**
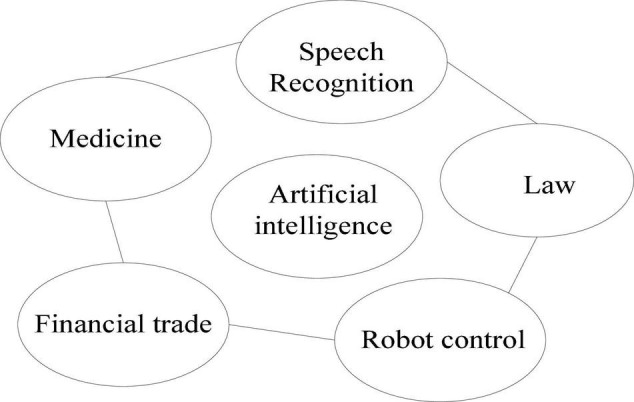
Main application areas of artificial intelligence.

In general, artificial intelligence systems mainly include learning systems, usage systems, and management systems. Among them, the technology in the learning system is the most critical part, because it must be learned before it can be used by the users of the system. In daily life, we can see many places where artificial intelligence technology is involved, such as P-picture software on mobile phones and intelligent assistants and autonomous driving technology. The fields of artificial intelligence research mainly include robotics, language recognition, image recognition, natural language processing, and expert systems. The basic application areas of artificial intelligence are shown in Figure 2.

Natural language processing is a discipline that uses computer technology to analyze, understand and process natural language. It studies various theories and methods that can realize effective communication between humans and computers using natural language. The goal is to make computers as intelligent as humans in understanding language, with the ultimate goal of bridging the gap between natural language and machine language.

The traditional oral English teaching mode cannot meet the individual requirements of students. Therefore, this paper combines artificial intelligence technology with deep learning algorithm to upgrade the traditional oral English teaching mode. Natural language processing is one of the basic methods of artificial intelligence, and it has been widely used in speech analysis and recognition. In order to meet the needs of students for oral training, we aim to carry out different oral language teaching modes according to the oral characteristics of different students, and carry out systematic simulation matching according to their inherent characteristics. Among them, the mathematical expression of analog matching is:


(1) $$Q_{n}=\sum_{u=1}^{b}w_{a}\,\left(k\right)\,P\,\left[k\right]$$

(2) $$P\,\left[k\right]=\sum_{n=0}^{N}x\,\left[n\right]\,e^{-i}$$

**FIGURE 3:**
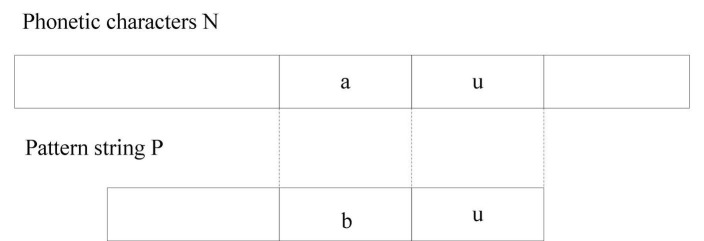
Simulation matching situation.

Among them, *p* represents the matching model, the matched part is *u*, *a* represents the phonetic characters, and the unmatched characters are marked as *b*. The situation of system simulation matching is shown in Figure 3.

In this process, different spoken language features require different functions for identification and matching, so the amount of computation and data is relatively large. In order to save resource storage space as much as possible and reduce the amount of computation, we have improved the original matching method and added a natural language processing model to it. In this model, semantic analysis is at the core of the successful operation of natural language processing, which can be basically expressed as:


(3) $$a=\sum_{i=1}^{r}\varpi_{i}\,x_{i}$$

(4) y=f⁢(a+γ)

In the above formula, *x* refers to the weight of the input value, _ϖ_ represents the weight of the input, _γ_ represents the threshold, and *y* refers to the output of neurons. It can be seen that neurons represent a many-to-one relationship, which can establish a bridge between input and output.

Based on this, we need to train and match the natural language processing to make it fit our needs. Among them, the training process of natural language processing is as follows:


(5) Eγ(v,h)=-∑iait-i∑jbjvhj

(6) $$v=-\sum_{i\,j}v_{i}\,w_{i\,j}\,h_{j}$$

(7) $$w\,\left(h\right)=\frac{1}{Z_{\theta }}\,\exp\left(-E_{\gamma }\,\left(v,h\right)\right)$$

Among them, *a* and *b* represent the number of layers and nodes in natural language processing, and *v* and *h* represent visible variables and invisible variables, respectively. Through this process, we can set up each layer and each node in natural language processing to serve us, and output qualified results in turn.

For invisible variables, each node is still independent of each other, that is, there is no connection between nodes, so it can be expressed as:


(8) $$P\,\left(h|v\right)=\prod_{j}P\,\left(h_{j}|v\right)$$

Further, we decompose the above formula so that it is connectable at *v*, then:


(9) $$P=\frac{1}{1+\exp\left(-\sum_{i}W_{i\,j}\,v_{i}-b_{j}\right)}$$

Among them, *P* represents the probability of achieving a perfect match within a specific step, and *v* and *b* represent the condition sets, respectively. Given the training samples and data, we can fit the training samples by setting up different sets of conditions.

In the process of data fitting, we need to establish a relatively complete evaluation system to describe the quality and level of oral expression. First, we need to extract the characteristic parameters of the test data and the corresponding data characteristics, and secondly, we need to perform pattern matching on the trained matching model, and then comprehensively compare the differences between the two.

Among them, the extraction process of data features is as follows:


(10) $$H\left(z\right)=1-ax{}^{-1}$$

(11) $$P_{m}=\sum_{m\in H}T\,\left(s\,\left(n\right)\right)\,\varpi \,\left(n-m\right)$$

Among them, *T* represents the mode transformation, and *s* stores the input data sequence. In the training process, our goal is to maximize the similarity, so the similarity function is defined as follows:


(12) $$L_{\theta }=\prod P\,\left(v^{i}\right)$$

However, for the above function, the continuous multiplication process involves more complex operations, so we decided to log it to reduce its time complexity and space complexity. The similarity is logarithmized as:


(13) $$\ln L_{\theta }=\ln\,\prod_{i}p\,\left(v^{i}\right)=\prod_{i}\ln p\,\left(v^{i}\right)$$

(14) $$\ln p\,\left(v^{i}\right)=\ln\,\sum_{h}\exp\left(-E\,\left(v,h\right)\right)$$

The gradient ascent method is generally used in the process of maximization, but its rising speed is too fast, which often breaks the boundary of the system and increases the amount of computation exponentially. So on this basis, the article sets a boundary for the matching model.


(15) $$a\,c\,f\,\left(\tau \right)=\sum_{i=0}^{n}s\,\left(i\right)\,s\,\left(i+r\right)$$

Among them, _τ_ represents the boundary factor, which can output the boundary value equidistantly and compare it with the matching maximum value in real time. At the same time, through this comparison, we can also discover the abnormality of the model in time, and make adjustments to the model in time.


$$d=100\times \left(\sum_{k=1}^{m-1}\left|d\,1_{k}-d\,2_{k}\right|\right)$$

Based on this, we can basically evaluate different levels of spoken English and give appropriate practice suggestions. In this process, model matching and evaluation methods based on natural language processing can help us to further understand the students’ spoken language and give professional suggestions. At the same time, it also overcomes the defects of traditional pattern matching and greatly reduces the matching time.

### Application of artificial intelligence in oral English teaching

**FIGURE 4:**
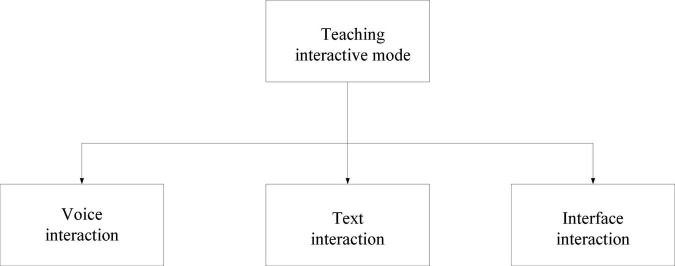
Artificial intelligence (AI) teaching interaction mode.

With the further integration and development of “Internet +,” cloud computing technology, big data analysis technology and artificial intelligence technology are widely used in the field of teaching (Guo and Niu, 2021). When deep learning is combined with other cognitive sciences and linguistics, it may be able to exert greater power to solve the problem of semantic understanding and bring true “intelligence.” At the same time, mobile phones and tablet computers have also brought certain opportunities for the development of digital classrooms. As we all know, the practicability of the subject of English is very strong. The learner must input a large number of vocabulary, and it is possible to improve the English ability after many times of thinking training. Beginners generally have a weak foundation, and the ability of oral expression can be improved quickly and greatly. However, beginners are generally in their teenage years, and their thinking is relatively active, and they are extremely sensitive to information and the Internet (Zou et al., 2022). Although deep learning has greatly improved the performance of natural language processing, the field is the science of language technology. It’s not about finding the best machine learning method, it’s still a linguistic problem at the core. Artificial intelligence technology spans many fields, and it mainly simulates human conscious activities and thinking processes (Seraj et al., 2021). Therefore, artificial intelligence technology can be used in the teaching of spoken English. And it relies on the information teaching platform to create a learning library to build a smart classroom model. This model integrates a series of functions such as the diagnosis of learning situation, the release of tasks, the correction of teachers’ homework, and the evaluation system (Opeifa et al., 2022). It can create an intelligent learning environment. This environment is characterized by playfulness, intelligence and aesthetics, while realizing the unity of individual teaching and customized teaching. It enhances the professional competence of teachers. A schematic diagram of the teaching interaction mode in the context of artificial intelligence is shown in Figure 4.

The application of artificial intelligence in modern education and teaching can not only enable students to master the basic knowledge and basic skills of majors, but also use artificial intelligence technology to optimize the quality of classroom teaching. It assists teachers in teaching better and improves the work efficiency of teachers. A complete AI-optimized teaching process is as follows: It should make full use of various resources in the information-based teaching platform, and teachers will then use systems such as the Correction Network to ask students questions. At this time, the AI intelligent learning situation diagnostic device can play its role. It extracts the keywords answered by students, and screens out some mistakes that students often make, such as improper vocabulary spelling, incomplete grammar knowledge, insufficient vocabulary, etc. The teacher then prescribes the right medicine for these problems, and requires students to use the word learning and oral practice APP to punch in, so as to achieve the effect of consolidation and improvement. The AI robot in the system can also conduct daily conversations and exchanges with students at any time (Qiao et al., 2021).

## Pattern matching of English teaching based on natural language processing

In order to study the oral English teaching mode in the actual situation, the article conducts a controlled experiment on three parallel classes of the seventh grade in a certain place. Among them, in order to detect the oral English of the tested students, we used the recording software CoolEdit to regularly record and sample them, and conduct regular English oral performance assessments for these three classes. Among them, the English speaking scores of the three classes before the start of the experiment are shown in Table 1.

**TABLE 1:** Statistical results of oral English scores.

Tested subjects	Class	Below 60 points	60–70 points	70–80 points	80 points or more
Oral English	Experimental class 1	2	3	13	13
	Experimental class 2	2	4	11	12
	Control class	1	5	10	15

Table 1 shows that there is little difference between the oral English scores of the experimental group and the control group. Among them, the number of students in the control class with a score of less than 60 in speaking is 1 less than that in the experimental class, and the number of students in the 60–70 score in the control class is 2 more than that in the experimental class. In particular, among the students with a score of 80 or above, there are 3 more students in the control class than in the experimental class. However, in the 60–80 score segment, the number of people in the control class and the experimental class is basically the same, between 15 and 16.

In the general assessment of oral English scores, different parts have different proportions, and the degree of difficulty is also different. This will also directly affect students’ basic performance and training methods. If we want to upgrade and adjust the teaching mode of oral English, then we must first adjust the various parts of the oral test. Among them, the basic situation of the proportion of each part in the English test is shown in Table 2.

**TABLE 2:** The proportion of each part in the English achievement test.

Question type	Percentage (%)	Actual score	Degree of difficulty
Oral pronunciation	35	42	5.2
Speed	20	24	6.0
Intonation	25	30	4.8
Precise	20	24	5.0

Table 2 shows that in the traditional oral English test, the investigation mainly focuses on the intonation of oral English and the accuracy of oral expression. Therefore, in the composition of oral English scores, intonation and oral pronunciation accounted for a relatively large proportion, as high as 35 and 25%, and their actual scores also reached 42 and 30 points. However, in terms of the actual difficulty coefficient of spoken English, the intonation difficulty coefficient of spoken English is relatively low, with a coefficient of 4.8, which is relatively difficult.

However, the traditional oral practice and assessment focus on general problems and cannot take into account the individual situation of students. Based on this, we use natural language processing to upgrade the traditional teaching mode, so that it can match different students’ oral English teaching mode suitable for him. To examine the practical utility of this method, we conduct a detailed study on the following aspects.

After adopting this method, we found a certain change in the number of students asking questions before class. In the traditional model, students do less self-thinking, so they can’t ask questions clearly. Under the existing model, students can more accurately find their own problems in spoken language, and the number of people who ask questions before class is shown in Table 3.

**TABLE 3:** Number of questions asked before class.

Class	Two minutes early	Six minutes ahead	Eight minutes ahead
Experimental class 1	1.333	2.333	2.5
Experimental class 2	1.5	2.233	2.4
Control class	0.333	1.333	2.333

Table 3 shows that in the 2 min before class, the number of questions asked by students in the experimental group increased rapidly year-on-year, with a maximum of 2 students. Also, as the time advances, the number of questions asked by each class is also increasing. In particular, when the time was advanced to 8 min, the number of people in the experimental class and the control class were basically the same, about 3 people. This shows that we cannot see the effectiveness of this model in improving spoken language only from the number of questions asked.

The grades can usually reflect the basic situation of students’ learning and the effectiveness of the teaching mode more intuitively. Therefore, we made a simple comparison of the mid-term and final grades of students in the three classes, and the results are shown in Tables 4, 5.

**TABLE 4:** Comparison of mid-term scores in oral English.

Class	Average score	Standard deviation	Variance
Experimental class 1	111.79	16.56	22.57
Experimental class 2	112.42	15.17	21.87
Control class	112.13	15.09	21.64

**TABLE 5:** Comparison of final grades of oral English.

Class	Average score	Standard deviation	Variance
Experimental class 1	113.31	10.77	19.17
Experimental class 2	113.19	10.74	19.07
Control class	113.11	11.07	20.30

Tables 4, 5 show that after a period of pattern training and matching, the students’ speaking scores have been basically improved. Among them, in the final exam, the average score of the experimental class was 112.42, which was higher than the 112.13 of the control class. This shows that pattern matching based on natural language processing is beginning to work (Guo and Zhu, 2022; Wang, 2022). But at the same time, we can also see that the standard deviation of the experimental class is much higher than that of the control class, which shows that the fluctuation of their scores is relatively large, and the dispersion of scores is relatively strong. At the end of the term, we found that the performance of the experimental class improved significantly year-on-year. The average score of the two classes was higher than that of the control class, and the variance was basically 10.7, which was significantly lower than the control class’s 11.07. This fully shows that after a period of training and learning, the teaching mode based on natural language processing has achieved remarkable results.

## Spoken English teaching mode under natural language processing

**FIGURE 5:**
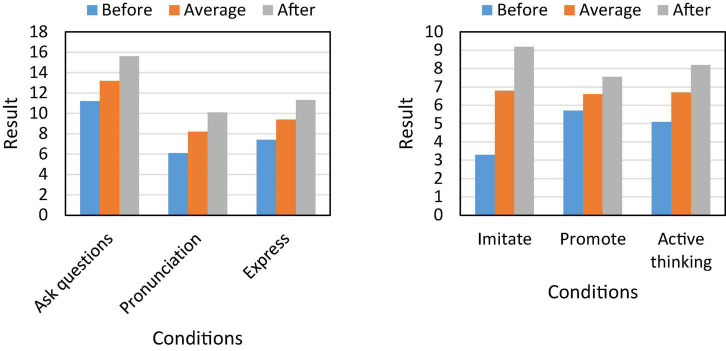
Students’ enthusiasm for learning oral English.

By setting up corresponding experimental classes and control classes, we can basically know that the performance of the students in the experimental class has been greatly improved. However, what aspects have the students in this class improved, and what attitudes they will hold toward the study of spoken English in the future, these are all waiting for the article to continue to study. To this end, based on the experiment, we continue to analyze the enthusiasm of students to learn oral English, and the results are shown in Figure 5.

Figure 5 shows that teaching pattern matching based on natural language processing can effectively improve students’ interest in oral exercise, allowing them to think at different levels and achieve progress. Among them, we can see that students’ enthusiasm for learning oral language has increased by 33.3% year-on-year, and their imitation ability has increased by 66.7%, which further promotes students to think actively and ask questions.

**FIGURE 6:**
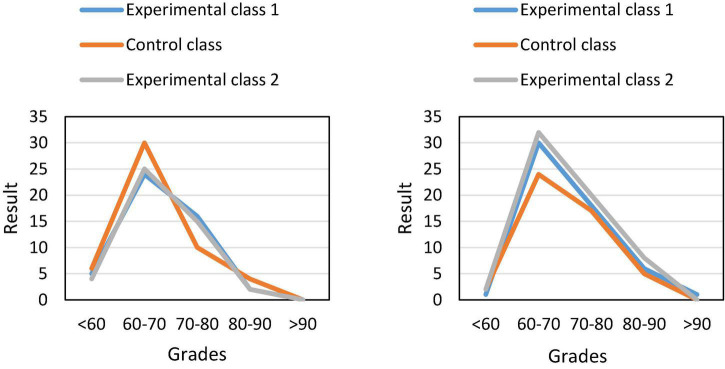
Segmentation of students’ midterm and final grades.

In the above experimental process, we only made a comprehensive comparison of the average grades of the three classes. However, the number of people in each of the fractional segments was not counted. In order to intuitively see the specific situation of students’ grades in different classes, we made statistics on the number of students in each score segment. The final result is shown in Figure 6.

Figure 6 shows that in the mid-term stage, the scores of the students in the control class accounted for a relatively heavy proportion in the 60–70 score segment, and the proportion of the students with low and high scores was relatively low, and the overall distribution was stable and even. After reaching the final stage, we found that the experimental class occupies a considerable proportion between the 60–80 grades. At the same time, the distribution of grades also tends to be stable, and no longer presents the previous discrete situation (Pan et al., 2020; Jiao and Liu, 2021).

**FIGURE 7:**
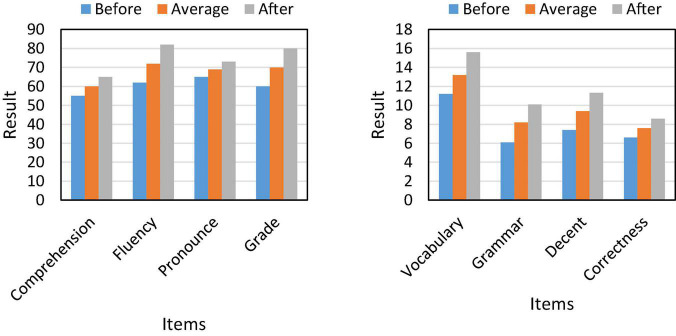
Students’ oral English ability.

Based on the above, we found that the teaching mode matching based on natural language processing can effectively improve students’ oral performance and make their learning process more active. Therefore, we studied the comprehensive ability of students’ oral English, and the results are shown in Figure 7.

The data in Figure 7 shows that the teaching mode based on natural language processing can improve students’ comprehensive ability of spoken English. And its comprehension increased by 19.7% year-on-year, and the fluency of spoken language increased to 86%. At the same time, the students’ vocabulary mastery has also increased by 16.1% year-on-year, and their mastery of spoken language has also risen to a higher level.

## Conclusion

Natural language processing is one of the important branches in the field of artificial intelligence, which can realize real-time processing of language signals, so this article introduces it into the teaching of spoken English. Based on the College English teaching model, this study first analyses the traditional teaching model and obtains its basic characteristics. On this basis, the article further introduces the natural language processing model in artificial intelligence, and then proposes a spoken English teaching model based on natural language processing. Experiments show that the oral English teaching mode based on natural language processing can effectively improve students’ comprehensive ability of oral English. However, due to time reasons, the article does not horizontally compare the oral English teaching modes under other technologies. In the future, the article will aim to explore the teaching mode under different technologies, hoping to provide reference for the reform and upgrading of the teaching mode.

## Data availability statement

The original contributions presented in this study are included in the article/supplementary material, further inquiries can be directed to the corresponding author.

## Author contributions

The author confirms being the sole contributor of this work and has approved it for publication.

## Conflict of interest

The author declares that the research was conducted in the absence of any commercial or financial relationships that could be construed as a potential conflict of interest.

## Publisher’s note

All claims expressed in this article are solely those of the authors and do not necessarily represent those of their affiliated organizations, or those of the publisher, the editors and the reviewers. Any product that may be evaluated in this article, or claim that may be made by its manufacturer, is not guaranteed or endorsed by the publisher.
